# Infection-related mortality after hematopoietic stem cell transplantation in a high antimicrobial resistance setting

**DOI:** 10.1007/s10096-026-05481-w

**Published:** 2026-04-22

**Authors:** Pelin İrkören, Sinem Civriz Bozdağ, Ümit Barbaros Üre, Nazlı Ataç, Füsun Can, Meltem Akay, Önder Ergönül

**Affiliations:** 1 Department of Infectious Diseases and Clinical Microbiology, Ünye State Hospital, Ordu, Türkiye; 2 Department of Hematology, Koç University School of Medicine, İstanbul, Türkiye; 3 Koç University İşbank Center for Infectious Diseases (KUISCID), İstanbul, Türkiye; 4 Department of Microbiology, Koç University School of Medicine, İstanbul, Türkiye; 5 Department of Infectious Diseases and Clinical Microbiology, Koç University School of Medicine, İstanbul, Türkiye

**Keywords:** Mortality, Bone marrow transplant, Bloodstream infections

## Abstract

**Background:**

Infectious complications remain a leading cause of mortality after hematopoietic stem cell transplantation (HSCT). We aimed to describe post-HSCT infections and their impact on survival in a center from a region with high antimicrobial resistance.

**Methods:**

We included 522 patients with hematological malignancies undergoing HSCT at Koç University Hospital between 2016 and 2024. Bloodstream and opportunistic infections within 100 days were analyzed. Infection-related mortality (IRM) was defined as death primarily attributable to infectious complications. Predictors of 30- and 100-day mortality were assessed using multivariate logistic regression, including demographic and hematologic features, bloodstream infections (BSIs), invasive aspergillosis (IA), and cytomegalovirus reactivation.

**Results:**

The most frequent diagnoses were non-Hodgkin lymphoma (36%), multiple myeloma (33%), and Hodgkin lymphoma (11%). Gram-negative bacteria (GNB) accounted for 73% of BSIs; *Escherichia coli* (31%) and *Klebsiella pneumoniae* (30%) were most frequent. Carbapenem resistance was highest in *Klebsiella pneumoniae* (67%), mainly due to OXA-48 carbapenemase (43%). Carbapenem-resistant *Klebsiella pneumoniae* (CRKP) bacteremia was associated with significantly reduced 100-day survival (log-rank *p* < 0.001). IA also significantly impaired survival in the overall cohort (log-rank *p* < 0.001). In the allogeneic subgroup (*n* = 139), 100-day mortality reached 27%, with IA occurring in 19% and CRKP bacteremia in 17%.

At 30 days, 25 patients (5%) had died; 24 of these deaths (96%) were attributed to infections. By day 100, 52 patients (10%) had died, and infections accounted for 42 deaths (80%).

**Conclusion:**

In a high antimicrobial resistance setting, CRKP bacteremia and invasive aspergillosis were major determinants of early post-HSCT mortality. These findings highlight the need for optimized empirical therapy, antimicrobial stewardship, and strengthened infection prevention strategies in this vulnerable population.

## Highlights

Infection-related mortality accounted for the majority of early deaths after HSCT in this high antimicrobial resistance setting.

Carbapenem-resistant *Klebsiella pneumoniae* bacteremia was an independent predictor of both 30-day and 100-day mortality.

*Pseudomonas aeruginosa* bacteremia and invasive aspergillosis were also associated with significantly reduced survival.

The OXA-48 gene was the most common molecular type of *Klebsiella pneumoniae*.

Invasive aspergillosis occurred in 12% of patients—particularly in the allogeneic group—and was strongly associated with prolonged neutropenia and corticosteroid use.

## Introduction

Hematopoietic stem cell transplantation (HSCT) is an effective treatment option for hematologic malignancies and immunodeficiency syndromes. However, infections become inevitable due to complications such as neutropenia, lymphopenia, hypogammaglobulinemia, and disruption of the cutaneous and mucosal barriers [1–5]. Infections can cause delayed hematopoietic recovery, graft rejection, prolonged hospitalization, increased healthcare costs, and decreased quality of life for patients undergoing HSCT [6]. Although immunological components show numerical recovery, functional abnormalities and infections can still occur in the case of graft-versus-host disease (GVHD) or persistent immunosuppression [7–9].

Both hematological and infectious factors influence mortality among HSCT recipients. Infection-related mortality (IRM) throughout the post-transplant period has been associated with various factors, including older age, advanced disease stage, type of transplant, stem cell source, and the number of transplantations [10, 11]. Bloodstream infections (BSIs) are the most common bacterial infections, with rates of 5–10% in autologous transplants and 20–50% in allogeneic transplants during the pre-engraftment period, primarily due to conditioning regimens that damage mucosal surfaces [12–17]. Central venous catheters (CVC), an insufficient immune response, and GVHD-related variables are the main risk factors for infection during the early post-engraftment period [12–15]. Infections caused by Gram-negative bacteria (GNB) are a major contributor to mortality, particularly during the pre-engraftment and early post-engraftment phases [18, 19]. Furthermore, infections with multidrug-resistant (MDR) GNB, delayed or inappropriate empirical antibiotic therapy, admission to the intensive care unit, and the presence of shock at onset have all been identified as significant predictors of increased mortality [14, 15]. The risk of invasive fungal infections (IFIs) continues in around 10% of patients who have poor graft function and receive high-dose steroids due to chronic GVHD [20, 21].

We aimed to assess how post-transplant infections affect survival outcomes in a setting in a high-antimicrobial-resistance region. We explored the incidence, risk factors, prevention, and treatment options for infections after HSCT. The results are expected to aid in developing effective strategies to prevent and treat infections, thereby reducing IRM in this high-risk group.

## Method

We included 522 patients with hematological malignancies who underwent autologous or allogeneic HSCT at the Koç University Hospital (KUH) Bone Marrow Transplantation Unit between November 2016 and July 2024. Patients under the age of 18, stem cell donors, and patients with non-hematological diseases were excluded.

In this retrospective study, demographic characteristics and medical histories were recorded by screening the KUH database (Nucleus ^®^). BSIs and other opportunistic infections were monitored for the first 100 days following HSCT. Infection-related mortality (IRM) was defined as death primarily attributable to infectious complications, as determined by comprehensive clinical assessment, rather than death in which infection was present but not considered the primary cause.

### *Antimicrobial prophylaxis and empirical therapy*

Fluoroquinolones were the most commonly used antibacterial prophylaxis during neutropenia. In selected high-risk patients, broader-spectrum agents were administered according to clinical assessment and local epidemiology.

Antifungal prophylaxis was risk-adapted and evolved during the study period. In the early years of the study, fluconazole was commonly used, including in some allogeneic recipients. In later years, mold-active agents—primarily posaconazole—became the preferred prophylactic strategy in allogeneic HSCT patients, in accordance with evolving institutional practice and emerging evidence.

Empirical therapy for neutropenic fever consisted of an antipseudomonal beta-lactam agent, with escalation based on clinical severity and local resistance patterns. Glycopeptides were added in cases of suspected catheter-related infection, severe mucositis, or hemodynamic instability. In patients with documented colonization by multidrug-resistant organisms, empirical antimicrobial therapy was individualized according to the previously identified pathogen and institutional resistance patterns.

### *Definitions of opportunistic infections*

*Invasive fungal infections (IFIs) were defined as the detection of fungal forms or fungal-related tissue damage in histopathological or microbiological samples of sterile areas; evidence of invasion of fungal infection from non-sterile areas by clinical, radiological, and/or serological tests. Invasive aspergillosis (IA) was classified according to EORTC/MSG criteria as possible, probable, or proven. The cut-off value for galactomannan positivity was determined as 1 ng/ml in serum and 0.7 ng/ml in bronchoalveolar lavage (BAL) fluid [22].

*Pneumocystis jirovecii pneumonia (PJP) was defined as the detection of positivity in lower respiratory tract samples (sputum, BAL) by polymerase chain reaction (PCR) or direct fluorescent antibody (DFA) and Giemsa staining of patients with clinical risk factors and radiologically compatible with PJP.

*Clostridioides difficile infection (CdI) was defined as *C. difficile* glutamate dehydrogenase (GDH) and C. difficile Toxin A&B or *C. difficile* PCR positivity in stool samples of patients with three or more watery stools per day.

*Cytomegalovirus (CMV) and Epstein-Barr Virus (EBV) Reactivations were defined if serum viral PCR levels were > 1000 copies/mL with other clinical factors [23, 24].

*BK Polyoma Virus (BKPyV) Reactivation was defined as the triad of detection of cystitis symptoms, macroscopic hematuria, and BKPyV DNA > 7 log10 copies/mL in urine or plasma viral load > 3–4 log10 copies/mL [25].

### *Microbiological assessment and molecular studies*

Blood cultures were incubated in the BacT/ALERT 3D (bioMérieux, France) automated system at the KUH Microbiology Laboratory for 7 days. In case of any positivity, matrix-assisted laser desorption/ionization time-of-flight mass spectrometry (MALDI-TOF MS) was used for identification.

Stored *Klebsiella pneumoniae* isolates from the archive (-80 °C freezer) of the KUH Microbiology Laboratory were retrieved (*n* = 34). After subculture on 5% sheep blood agar and incubation for 24–48 h, 23 isolates showed viable growth, which was then used for molecular analysis. Bacterial colonies were suspended in sterile water, heated at 95 °C for 15 min, then centrifuged at 20,000 g for 5 min. The supernatant was utilized to perform multiplex PCR amplification of the OXA-48-like carbapenemase, *Klebsiella pneumoniae*-like carbapenemase (KPC-1), and New Delhi metallo-beta-lactamase (NDM) carbapenemase genes. PCR products were examined using agarose gel electrophoresis.

### *Statistical methodsStatistical methods*

For comparison of categorical and proportional variables, Pearson chi-square and Fisher-Exact tests; for the continuous variables, Student’s t-test or Mann–Whitney U test according to distribution. Overall survival within the first 100 days after HSCT was assessed using Kaplan–Meier survival analysis, and survival differences between groups were evaluated with the log-rank test. Survival time was determined from the date of transplantation until death, with patients censored at day 100 if they survived.

Variables including age, gender, primary hematological diagnosis, relapsed/refractory (R/R) disease status, type and number of transplantations, engraftment days, bloodstream infection pathogens, invasive aspergillosis, CMV reactivation, GVHD, corticosteroid use, and ICU admission were included in univariate analyses for mortality. The first BSI attack of the patients was accounted.

Variables considered clinically relevant or statistically significant were subsequently entered into multivariate logistic regression models, and backward selection was performed for the 30-day and 100-day mortality. In addition, a subgroup analysis was performed among allogeneic HSCT recipients, and a separate multivariate model was constructed to define predictors of 100-day mortality in this higher-risk population. For statistical significance, the p-value was set as < 0.05, and STATA-BE 17.1 and SPSS software packages were used.

## Results

The mean age of 522 HSCT recipients was 51, and 284 (55%) were males. The primary hematological malignancies were non-Hodgkin lymphoma (36%), multiple myeloma (33%), Hodgkin lymphoma (11%), and acute myeloid leukemia (9.5%) (Table 1). Of the transplants, 383 (73%) were autologous, and 139 (27%) were allogeneic. GVHD was developed in 49 (%35) patients among the allogeneic transplant group. After transplantation, 114 (22%) patients required systemic steroid therapy, which was defined as a steroid equivalent to 20 mg/day methylprednisolone for more than 20 days.

**Table 1:** Demographic and clinical features of the patients

	*n* = 522 (%)
Mean Age (SD; min-max)	51 (14.1;18–75)
Male	284 (55)
Systemic Disorders	
Hypertension	84 (16)
Diabetes Mellitus	64 (12)
Chronic Renal Failure	21 (4)
Coronary Artery Disease	20 (4)
Solid Cancer	11 (2)
Chronic Obstructive Lung Disease	9 (2)
Renal Transplant	5 (1)
History of Infectious Diseases	
Human Immunodeficiency Virus Infection	2 (0.4)
Past Invasive Fungal Disease	37 (7)
0–30 days	9 (1.7)
31–100 days	9 (1.7)
> 100 days	20 (5)
Primary Hematological Disease	
Non-Hodgkin Lymphoma	186 (36)
Multiple Myeloma	170 (33)
Hodgkin Lymphoma	58 (11)
Acute Myeloid Leukemia	50 (9.5)
Acute Lymphoblastic Leukemia	28 (5)
Myelodysplastic Syndrome	21 (4)
Chronic Lymphocytic Leukemia	3 (0.5)
Chronic Myeloid Leukemia	3 (0.5)
Aplastic Anemia	3 (0.5)
Relapse/Refractory Disease	184 (35)
Number of Transplantation:	
1	470 (90)
2	52 (10)
Type of Transplantation:	
Autologous	383 (73)
Allogeneic	139 (27)
Neutrophil Engraftment Day (SD; min-max)	11.6 (4.1; 5–40)
Platelet Engraftment Day (SD; min-max)	13.8 (7.6; 4–80)
Mean Hospitalization Days (SD; min-max)	31.5 (16.3; 15–145)

Febrile neutropenia episodes were seen in 489 (94%) patients during the pre-engraftment period. Only 144 BSIs were documented microbiologically during the 100-day follow-up. Of the BSI episodes, 122 (84%) were classified as primary BSIs, associated with mucositis or CVC use. Gram-negative bacteria (73%) were detected at a higher rate than Gram-positive bacteria (GPB) (24%) in blood cultures. Among the bacterial agents, the most common were *Escherichia coli* (31%) and *Klebsiella pneumoniae* (30%) (Table 2). The phenotypic carbapenem resistance rate of *Klebsiella pneumoniae* isolates was 67% (29/43). Among *Klebsiella pneumoniae* isolates (*n* = 23), further molecular analysis revealed that the OXA-48 gene was detected as positive in 43% (10/23), the KPC-1 gene was detected as positive in 26% (6/23), and the NDM-1 gene was detected as positive in 9% (2/23).

**Table 2:** Distribution of bloodstream infection agents and phenotypic resistance patterns

	Total Number *n* = 144 (%)	Phenotypic Resistance	Resistance Rates *n* (%)
Gram-Negative Bacteria			
*Escherichia coli*	45 (31)		*n* = 45 (%)
		Fluoroquinolone	36 (80)
		ESBL	15 (33)
		Piperacillin tazobactam	11 (24)
		Carbapenem	2 (4)
		Amikacin	3 (6)
*Klebsiella pneumoniae*	43 (30)		*n *= 43 (%)
		Fluoroquinolone	34 (79)
		ESBL	34 (79)
		Piperacillin tazobactam	35 (82)
		Carbapenem	29 (67)
		Amikacin	5 (12)
*Pseudomonas aeruginosa*	12 (8)		*n* = 12 (%)
		Fluoroquinolone	6 (50)
		Piperacillin tazobactam	5 (42)
		Carbapenem	3 (25)
		Amikacin	2 (16)
*Stenotrophomonas maltophilia*	4 (3)		
Gram-Positive Bacteria			
Coagulase (-) *Staphylococcus* spp.	19 (13)	Methicillin	*n* = 19 (%) 12 (63)
*Enterococcus* spp.	9 (6)		*n* = 9 (%)
		Ampicillin	8 (88)
		Vancomycin	1 (11)
*Streptococcus* spp.	8 (5)		
*Staphylococcus aureus*	1 (0.7)	Methicillin	0
*Candida spp*.	5 (4)		
*Fusarium spp.*	1 (0.7)		
Others	8 (5)		
Polymicrobial			
2	9 (6)		
3	1 (0.7)		

Vancomycin-resistant *Enterococcus faecium* (VRE) was isolated in one patient. Nosocomial infection agents like *Acinetobacter baumannii* or methicillin-resistant *Staphylococcus aureus* (MRSA) were not isolated during the study period. Fungal agents were detected in %5 (*n* = 6) of the BSI episodes. Among polymicrobial BSIs, two bacteria were isolated in nine (1.7%) episodes, and three bacteria were isolated in one (0.2%) episode.

Invasive aspergillosis was detected in 66 (12%) patients in the 100-day follow-up. The incidence was higher in allogeneic recipients (19.5%, *n* = 27) compared to autologous recipients (10%, *n* = 39) (Table 3). The definite diagnosis was made only in two patients by demonstrating *Aspergillus spp.* hyphae pathologically. Statistical analysis showed that the risk of IA was significantly associated with prolonged neutropenia (OR 3.4, 95% CI: 1.894–6.293, *p* < 0.001), systemic steroid treatment was administered (OR 3.9, 95% CI: 2.322–6.821, *p* < 0.001), and allogeneic transplantation (OR 2.1, 95% CI: 1.245–3.630, *p* = 0.006). Past IFI was not found to be a significant risk factor for the development of IA (OR 0.4; 95% CI 0.12–1.48; *p =* 0.164).

**Table 3:** Opportunistic infections detected within 100 days after HSCT, including invasive aspergillosis and viral reactivations

	Total Number *n* = 522 (%)	Allogeneic HSCT *n* = 139 (%)	Autologous HSCT *n* = 383 (%)
Invasive Aspergillosis	66 (12)	27 (19.5)	39 (10)
Possible	53 (80)	22 (16)	31 (8)
Probable	11 (16)	4 (3)	7 (2)
Definite	2 (3)	1 (0.7)	1 (0.2)
Cytomegalovirus Reactivation	99 (19)	77 (55)	22 (6)
BKyP Virus Reactivation	25 (5)	25 (18)	0
Ebstein-Barr Virus Reactivation	5 (1)	4 (3)	1 (0.2)
*Pneumocystis jirovecii*	4 (0.7)	3 (2)	1 (0.2)
*Clostrodiodes difficile*	17 (3)	7 (5)	10 (3)

Cytomegalovirus was the most frequently detected viral reactivation (Table 3). PJP diagnosis was made in 4 patients based on microbiological, radiological, and clinical features. *Pneumocystis jirovecii* PCR was positive in 2 patients, and *Pneumocystis jirovecii* DFA and Giemsa staining were positive in 2 patients with bilateral central ground glass images on thoracic computed tomography. *C. difficile* infection was detected in 14 (2.6%) of the patients diagnosed with clinical enterocolitis (*n* = 64, 12%).

Overall mortality rates were 5% (*n* = 25) within 30 days and 10% (*n* = 52) within 100 days after HSCT. When transplant type was evaluated, the allogeneic group had a significantly greater mortality rate (12% at 30 days and 27% at 100 days) than the autologous group (2% and 3.6%, respectively). Disease-related factors and prolonged engraftment periods were identified as significant non-infectious factors for mortality (Table 4).

**Table 4:** Univariate analysis of risk factors for 30-day and 100-day mortality after HSCT

	30 days			100 days		
	OR	95% CI	*p*	OR	9%5 CI	*p*
Age > 60	1.3	0.584–2.954	5.508	1.1	0.630–2.030	0.678
Male	2.7	1.088–7.058	0.026	1.2	0.706–2.267	0.427
Hematological Diagnosis						
Acute Myeloid Leukemia	1.2	0.534–2.763	0.641	1.1	0.634–2.064	0.654
Acute Lymphoid Leukemia	0.1	0.039–0.730	0.017	0.1	0.341–0.361	< 0.001
Non-Hodgkin Lymphoma	0.3	0.426–2.422	0.271	0.4	0.139–1.532	0.207
Myelodysplastic Syndrome	0.8	0.180–3.556	0.784	2.9	1.412–6.225	0.004
Hodgkin Lymphoma	3.7	1.194–11.798	0.024	4.09	1.702–9.827	0.002
Aplastic Anemia	7.5	2.503–22.562	< 0.001	3.01	1.058–8.160	0.039
Chronic Lymphoid Leukemia	-	-	-	4.5	0.408–51.484	0.217
Chronic Myeloid Leukemia	-	-	-	-	-	-
Multiple Myeloma	10.3	0.903-117.744	0.060	4.5	0.408–51.484	0.217
Relapse/Recurrent Disease	2.9	1.280–6.619	0.008	2.3	1.317–4.185	0.004
Number of Transplant	3.9	1.548–9.853	0.008	2.4	1.135–5.182	0.022
1						
2						
Type of Transplant	6.5	2.750-15.509	< 0.001	9.9	5.171–19.016	< 0.001
Autologous						
Allogeneic						
Neutrophil Engraftment > 15 days	46.9	15.491–142.200	< 0.001	15.8	8.353–30.147	< 0.001
Platelet Engraftment > 15 days	39.9	9.285-172-241	< 0.001	13.3	6.730-26.411	< 0.001
Gram Negative Bacteremia						
* E. coli*	4.1	1.564–11.107	0.004	2.9	1.368–6.396	0.006
* K. pneumoniae*	9.7	3.964–24.182	< 0.001	6.3	3.142–13.032	< 0.001
* P. aeruginosa*	7.1	1.361–37.202	0.020	7.03	2.148–23.042	0.001
Gram Positive Bacteremia	1.22	0.276–5.411	0.791	1.1	0.399–3.485	0.764
Invasive Aspergillosis	4.9	2.068–11.700	< 0.001	4.1	2.177–7.998	< 0.001
CMV Reactivation	1.4	0.315–6.243	0.656	2.5	1.368–4.723	0.003
GVHD	0.4	0.055–3.166	0.715	1.7	0.726–4.067	0.218
Steroid Treatment	7.2	3.106–16.867	< 0.001	9.2	4.954–17.115	< 0.001
ICU Admission	-	-	< 0.001	196.5	46.280-834.486	< 0.001

Abbv: *E. coli* *Escherichia coli*, *K. pneumoniae* *Klebsiella pneumoniae*, *P. aeruginosa* *Pseudomonas aeruginosa*, *CMV* Cytomegalovirus, *GVHD* Graft-versus-Host Disease, *ICU* Intensive Care Unit

**Fig. 1:**
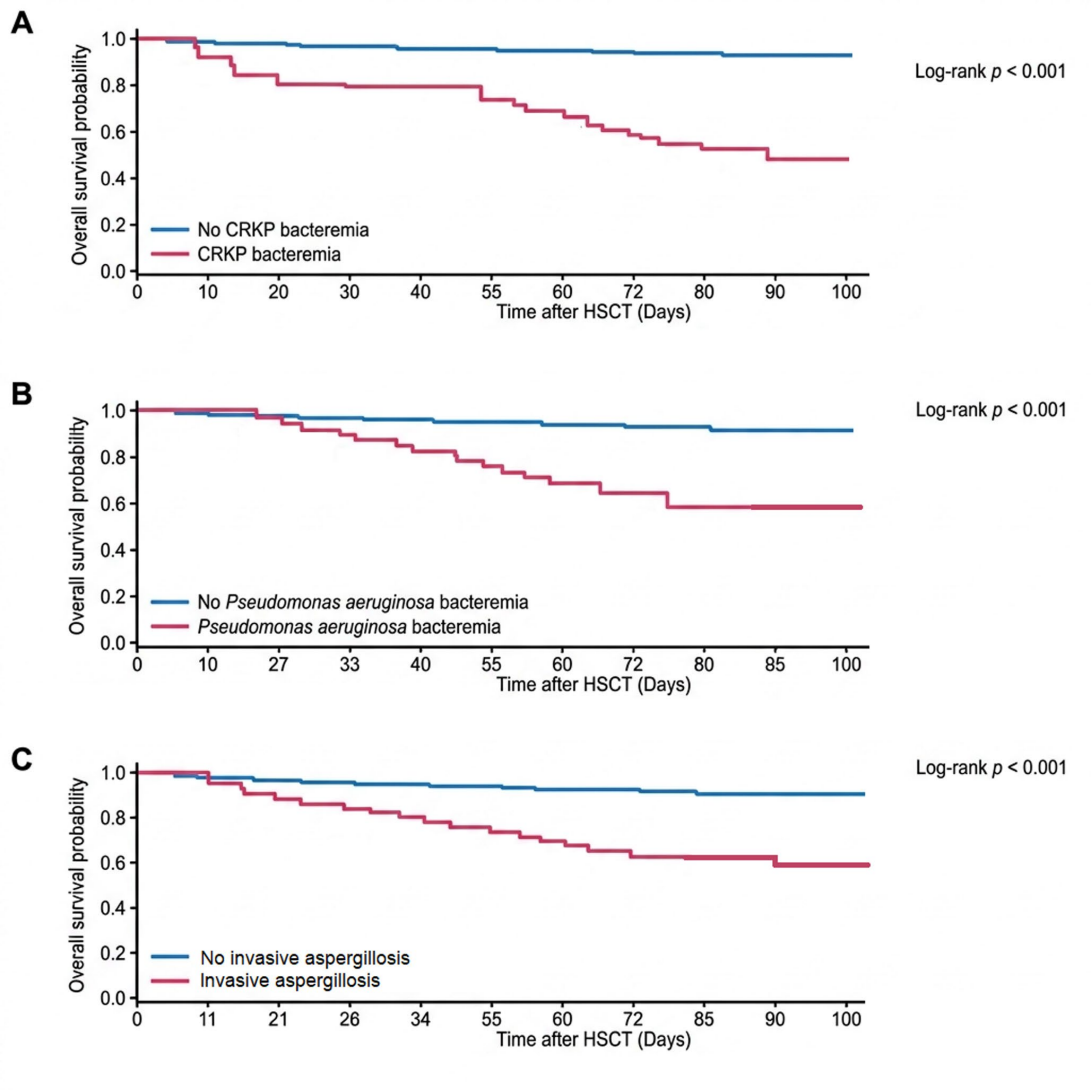
Kaplan–Meier curves showing 100-day overall survival stratified by (**A**) carbapenem-resistant *Klebsiella pneumoniae* (CRKP) bacteremia, (**B**) *Pseudomonas aeruginosa* bacteremia, and (**C**) Invasive aspergillosis, where both pathogens were associated with significantly reduced survival (log-rank *p <* 0.001 for both comparisons)

Infection-related mortality was notably high during the pre-engraftment period. Among the 25 deaths observed within 30 days, 24 (96%) were infection-related, and by day 100, 42 of 52 deaths (80%) were attributed to infections. Gram-negative bloodstream infections and IA emerged as the leading contributors to IRM. In Kaplan–Meier analysis, CRKP bacteremia and *Pseudomonas aeruginosa* bacteremia were both associated with markedly reduced 100-day survival (log-rank *p* < 0.001) (Fig. 1). Invasive aspergillosis also remained independently associated with 100-day mortality (OR 3.4, 95% CI 1.58–7.49; *p* = 0.002).

Cytomegalovirus reactivation was associated with increased 100-day mortality in univariate analysis (OR 2.5, 95% CI 1.37–4.72; *p* = 0.003), although this association did not remain significant in multivariate analysis.

Among allogeneic recipients (*n* = 139), 38 patients (27%) died within 100 days. IA occurred in 19% (*n* = 27) and *Klebsiella pneumoniae* bacteremia in 17% (*n* = 24) of this subgroup. In multivariate analysis restricted to allogeneic HSCT recipients, CRKP bacteremia remained independently associated with mortality (OR 5.64, 95% CI 1.91–16.63; *p* = 0.002), whereas IA did not retain independent significance.

In the overall multivariate model, primary disease status, allogeneic transplantation, IA, and CRKP bacteremia were independently associated with 100-day mortality (Table 5).

**Table 5:** Multivariate logistic regression analysis of predictors of mortality

	30 days			100 days		
	OR	95% CI	*p*	OR	95% CI	*p*
Age > 60	2.6	0.939–7.324	0.066	1.8	0.895–3.654	0.098
Male	2.9	1.038–8.534	0.042	1.08	0.548–2.154	0.811
R/R Disease	3.4	1.332–9.0451	0.011	2.2	1.167–4.505	0.016
Allogeneic Transplant	5.7	2.050-16.104	0.001	10.4	4.673–23.164	< 0.001
*E. coli* Bacteremia	3.6	1.045–12.933	0.042	2.5	0.987–6.519	0.053
*K. pneumoniae* Bacteremia	7.07	2.330-21.486	0.001	3.8	1.617–8.966	0.002
*P. aeruginosa* Bacteremia	7.6	1.229–47.524	0.029	3.8	0.957–15.399	0.058
Invasive Aspergillosis	4.8	1.743–13.443	0.002	3.4	1.576–7.485	0.002
CMV Reactivation	0.5	0.103–3.385	0.556	0.5	0.239–1.211	0.135

*Abbv*: *R/R* Relapse/Refractory, *E. coli* *Escherichia coli*, *K. pneumoniae* *Klebsiella pneumoniae*, *P. aeruginosa* *Pseudomonas aeruginosa*, *CMV* Cytomegalovirus

## Discussion

Hematopoietic stem cell transplantation remains a cornerstone in the management of hematological malignancies; however, the early post-transplant period continues to be marked by considerable infectious risk. In our cohort, overall mortality at 100 days was 10%, with allogeneic recipients having significantly higher rates (27%) than autologous recipients (3.6%), underlining the two populations’ distinct risk profiles. These mortality rates were slightly higher than those reported in the literature—approximately 3.4% for autologous and 8.3% for allogeneic transplants at 100 days [10, 19], likely reflecting the high burden of antimicrobial resistance in our setting.

Infection-related mortality accounted for the majority of early deaths. Importantly, IRM was defined as death primarily attributable to infectious complications based on a comprehensive clinical assessment. Notably, infections accounted for the majority of early deaths, particularly during the pre-engraftment phase, underscoring the vulnerability of patients in this period of profound immunosuppression. However, IRM should be interpreted with caution. As emphasized by McDonald et al. [26], early post-transplant deaths are frequently multifactorial. Relapse, graft failure, organ toxicity, graft-versus-host disease, and underlying comorbidities may all contribute to clinical deterioration, even when infection is present. In many cases, infection may represent the terminal event within a complex clinical course rather than the sole underlying cause of death. Consistent with previous reports [11], relapsed or refractory disease and allogeneic transplantation were independently associated with higher mortality in our cohort, further highlighting the cumulative impact of disease burden and treatment-related immunosuppression. Taken together, these findings suggest that while infections play a central role in early mortality, they often act within a broader network of transplant-related risk factors.

In terms of IRM, bloodstream infections, particularly those caused by GNB, were strongly associated with mortality, conferring a 4.1- to 9.7-fold increase in risk, consistent with prior data reporting up to a 16-fold increase [19]. In a long-term cohort, where BSIs after HSCT were screened for 25 years, a slight epidemiologic change was reported with the decline in the GPB and a rise in the GNB with extended-spectrum beta-lactamases (ESBL) production and multidrug resistance [27]. More recent data from Türkiye supported this trend, with a predominance of MDR-GNB isolated from febrile neutropenic patients [28].

The World Health Organization has similarly highlighted carbapenem-resistant *Acinetobacter baumannii*, *Pseudomonas aeruginosa*, and *Enterobacterales*—particularly CRKP and ESBL-producing *E. coli*—as priority pathogens in its 2024 list [29, 30]. In our study, 73% of the bacteremia agents were GNB, with *Escherichia coli* (31%) and *Klebsiella pneumoniae* (30%) as the most common. Alarmingly, carbapenem resistance was found in 67% of *K. pneumoniae* isolates, which corresponded with significantly increased mortality at both 30- and 100-day post-transplant.

**Fig. 2:**
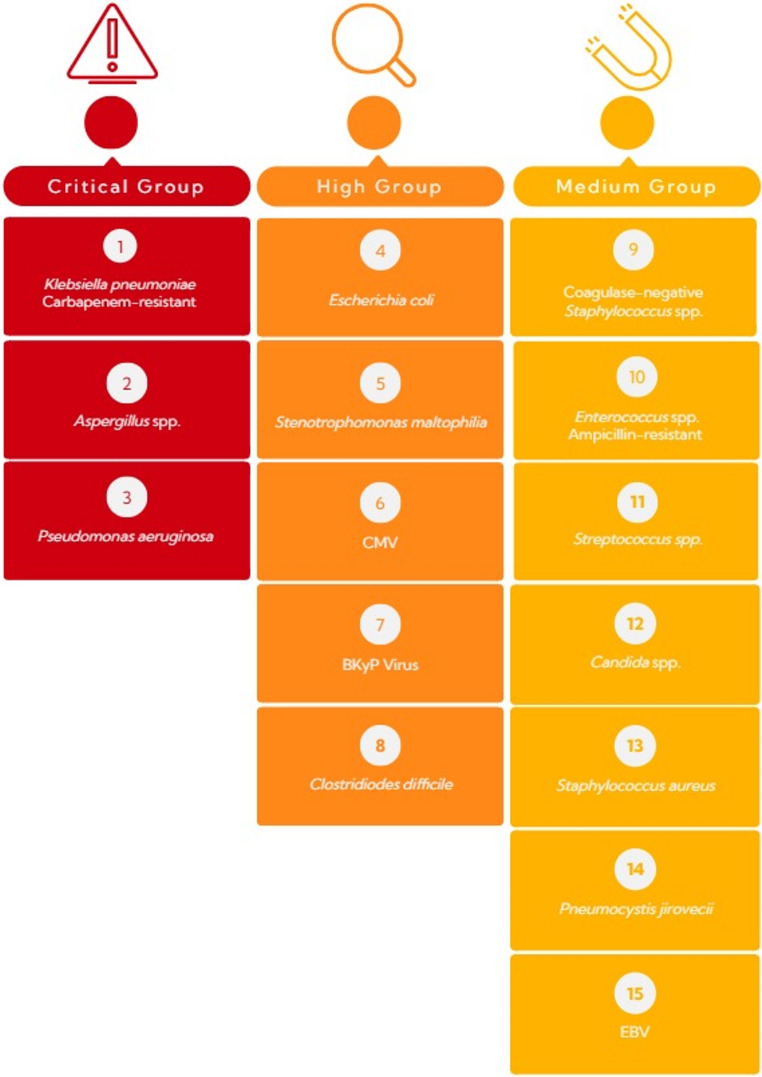
Institutional priority list of infections after hematopoietic stem cell transplantation

Based on our analysis, we prepared a priority list in terms of frequency and mortality rates of the infections (Fig. 2). Our eight-year surveillance categorized *Klebsiella pneumoniae*, *Pseudomonas aeruginosa*, and IA as very critically high-risk pathogens for mortality. *Escherichia coli* was classified as a high-risk pathogen due to its low carbapenem resistance and effective treatment options, while GPB and other opportunistic infections were classified as moderate-high-risk pathogens for mortality. These factors likely contributed to the poorer survival observed in patients with CRKP bacteremia.

Carbapenem-resistant *Klebsiella pneumoniae* bacteremia emerged as a critical determinant of survival. Türkiye is recognized as an endemic region for the OXA-48 type of carbapenemase, which poses substantial therapeutic challenges. Molecular analyses in our study confirmed OXA-48 as the predominant carbapenemase gene in CRKP isolates. A multicenter Turkish study demonstrated that ceftazidime-avibactam therapy significantly reduced mortality compared to conventional carbapenem treatments in OXA-48-positive *K. pneumonia*e bloodstream infections [31]. Although empirical treatment strategies were guided by international recommendations and local resistance patterns, CRKP bacteremia may not always have been adequately covered by first-line regimens. The limited availability and reimbursement restrictions of ceftazidime–avibactam during much of the study period likely contributed to delayed access to optimal therapy in selected cases. Another study revealed that initiating ineffective empirical therapy in bacteremia is linked to an increase in 30-day mortality [18]. Regarding this, it is recommended to develop empirical treatments tailored to institution-specific resistance profiles and to promote rational antimicrobial stewardship [32]. During much of our study period, access to ceftazidime–avibactam was limited and subject to reimbursement restrictions, potentially delaying optimal therapy, making *K. pneumoniae* bacteremia a significant factor negatively impacting survival in HSCT patients.

Invasive aspergillosis remains one of the most serious infectious complications following HSCT, significantly contributing to morbidity and mortality. In our cohort, IA developed in 12% of patients within the first 100 days post-HSCT, with a higher incidence in the allogeneic transplant group (19.5%) compared to the autologous group (10%). This is in line with previous studies reporting IA incidence ranging from 5% to 20% in allogeneic transplant recipients and around 2–8% in autologous HSCT, depending on risk factors and diagnostic criteria [33]. The pre-engraftment period and impaired cellular immunity create a window of vulnerability for IFIs, where allogeneic transplantation, active leukemia, previous IFI, and severe GVHD are recognized risk factors [34]. In our study, prolonged neutropenia and systemic corticosteroid use increased the risk of IA, highlighting their central role as predisposing factors. These findings are consistent with large cohort studies, which indicate that both neutropenia and immunosuppressive therapies are major risk factors for IA in HSCT recipients [35]. Prior IFI was not a significant risk factor for IA or mortality in our cohort, supporting evidence that previous fungal infections should not preclude transplantation when appropriate secondary prophylaxis is employed [36]. Despite its relatively low frequency, IA is associated with high mortality rates exceeding 50% in high-risk populations [37]. In our cohort, IA also significantly impaired survival, with Kaplan–Meier analysis demonstrating markedly reduced 100-day survival among affected patients (log-rank *p* < 0.001). IA occurred predominantly in allogeneic recipients, consistent with the recognized impact of prolonged neutropenia, impaired cellular immunity, and corticosteroid exposure. Although the predominance of possible IA cases according to EORTC/MSG criteria, the survival impact remained clinically meaningful. When restricted to probable/proven IA cases, the survival difference did not reach statistical significance, likely due to limited sample size.

Infection control practices also play a crucial role in limiting the spread of resistant pathogens and improving outcomes. Key interventions—such as hand hygiene, contact precautions, active surveillance cultures, environmental disinfection, and antimicrobial stewardship—have demonstrated efficacy in reducing mortality and antimicrobial consumption in patients with hematological malignancies [38, 39]. A prospective intervention study demonstrated that the implementation of rational antimicrobial stewardship programs significantly reduced mortality and overall antimicrobial drug use in patients with hematological malignancies due to episodes of febrile neutropenia [40]. Another systematic review supports that preventive isolation measures, including optimized ventilation and adherence to contact precautions, significantly decrease 30-day all-cause mortality [41]. A notable strength of our center is the absence of highly resistant pathogens such as *Acinetobacter baumannii* and MRSA in our patient population, reflecting sustained infection control efforts.

## Limitations

This study has several limitations. As a retrospective analysis based on data extracted from an electronic database, it may be subject to incomplete or inconsistent documentation. Clinical decisions and definitions relied on the judgment of treating physicians at the time, which may have introduced variability. In addition, the interpretation of radiological findings may have evolved, potentially affecting diagnostic accuracy.

Molecular analyses of bacterial isolates were performed retrospectively, which may have introduced selection bias and limited the availability of all causative strains for testing. Furthermore, the majority of invasive aspergillosis cases were classified as possible according to EORTC/MSG criteria, which may have led to an overestimation of the true incidence of proven/probable invasive fungal infections.

As a single-center study, the generalizability of our findings to other transplant centers, particularly those with different resistance patterns, may be limited. Finally, survival analyses were restricted to a 100-day follow-up, and longer-term outcomes were not assessed.

## Conclusion

Infections remain a major threat to early survival after HSCT, particularly in high antimicrobial resistance settings. In our cohort, multidrug-resistant Gram-negative bloodstream infections—especially carbapenem-resistant *Klebsiella pneumoniae*—emerged as key determinants of early mortality. Invasive aspergillosis also significantly impaired survival in the overall cohort, although its impact varied across transplant subgroups.

These findings underscore the importance of optimized empirical therapy tailored to local resistance profiles, improved access to effective antimicrobials, and strengthened infection prevention strategies in transplant centers operating in high-resistance environments.

## Data Availability

No datasets were generated or analysed during the current study.

## Abbrevetion

ESBL: Extended spectrum beta lactamase
